# Aftereffects of Spectrally Similar and Dissimilar Spectral Motion Adaptors in the Tritone Paradox

**DOI:** 10.3389/fpsyg.2018.00677

**Published:** 2018-05-08

**Authors:** Stephanie Malek, Konrad Sperschneider

**Affiliations:** ^1^Psychology Department, Martin Luther University Halle-Wittenberg, Halle, Germany; ^2^Psychology Department, Leipzig University, Leipzig, Germany

**Keywords:** tritone paradox, pitch perception, aftereffects, spectral motion detector, Shepard tones

## Abstract

Shepard tones consist of octave-spaced components, whose amplitudes are generated under a fixed bell-shaped spectral envelope. They are well defined in pitch chroma, but generate octave confusions that in turn can produce ambiguities in the perceived relative pitch heights when their chromas are exactly a tritone apart (the tritone paradox). This study examined the effects of tonal context on relative pitch height judgments using adaptor sequences followed by target sequences (pairs of Shepard tones of different chromas separated by a tritone). Listeners judged whether the second target Shepard tone was higher or lower than the first. Adaptor sequences consisted of rising or falling scales (43 s at the beginning of each block, 4 s before each target sequence). Two sets of Shepard tones were used for adaptors and targets that were generated under spectral envelopes centered at either A3 (220 Hz) and C6 (1,046 Hz). Pitch direction judgments (rising vs. falling) to spectrally consistent (A3–A3, C6–C6) and inconsistent (A3–C6, C6–A3) adaptor-target combinations were studied. Large significant contrastive aftereffects (0.08–0.21 change in fraction of pitch direction responses) were only found for the Shepard tones that were judged as higher in the control condition (judgments about the target sequences without adaptor sequences) for the consistent adaptor-target conditions (A3–A3, C6–C6). The experiments rule out explanations based on non-sensory decision making processes. Possible explanations in terms of perceptual aftereffects caused by adaptation in central auditory frequency-motion detectors are discussed.

## 1. Introduction

**Figure 1:**
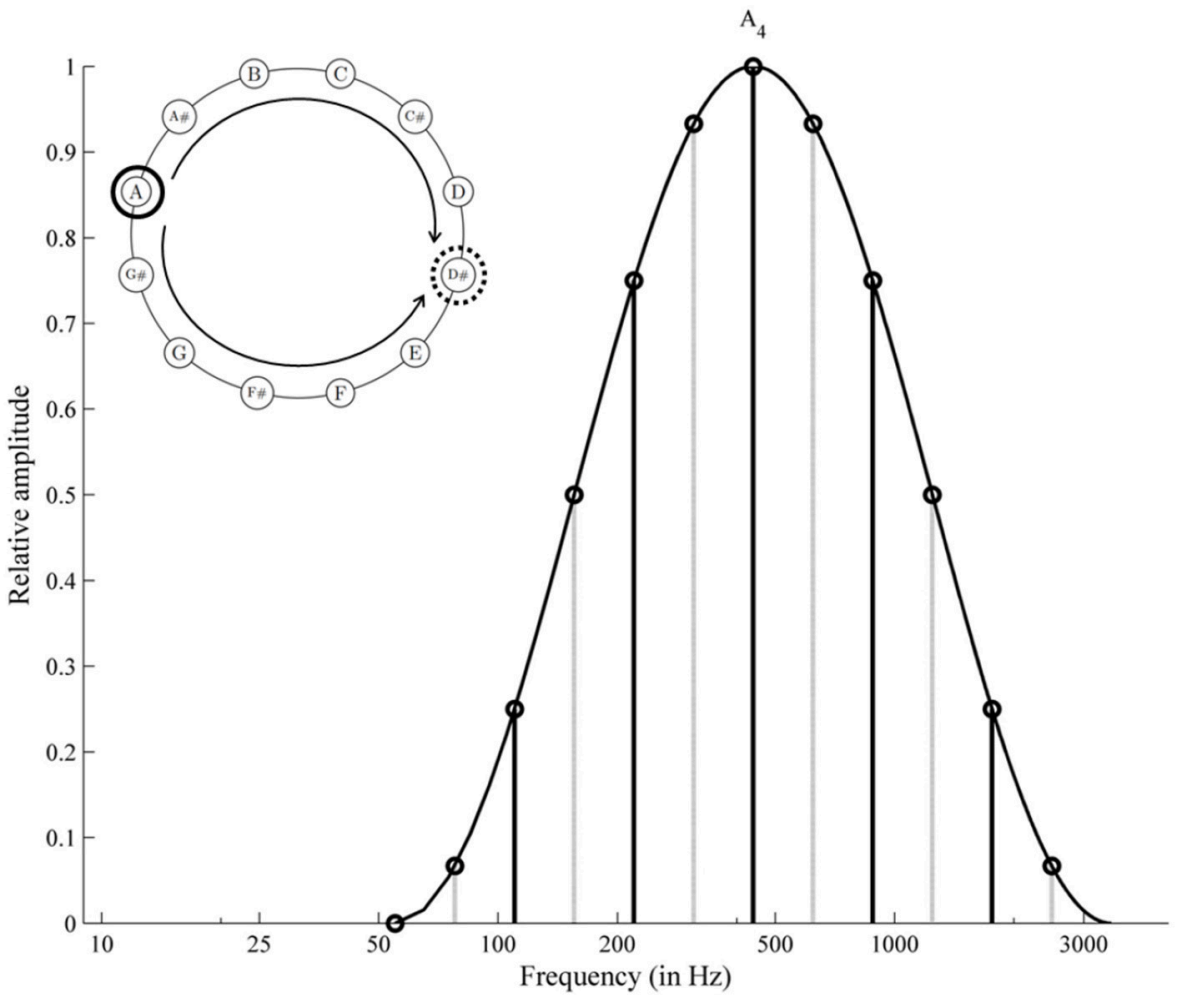
Frequency spectrum of two Shepard tones forming a tritone. The clockwise distance equals the counter-clockwise distance on the pitch-class circle; the octave-spaced frequency components generated under the cosine envelope centered at *A*_4_.

**Figure 2:**
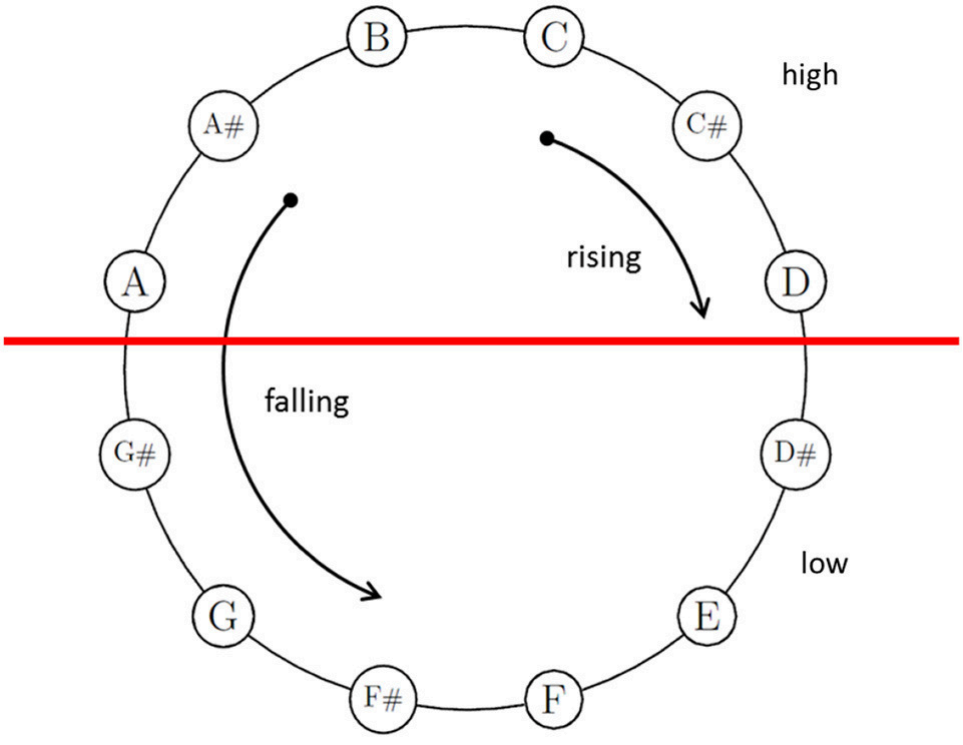
Shepard tones arranged in the pitch-class circle. Tritone paradox: Shepard tones in the upper half (*A*, *A#*, *B*, *C*, *C#*, *D*) are judged as higher than those of the lower half (*D#*, *E*, *F*, *F#*, *G*, *G#*). Proximity principle: When the distance between two pitch classes is clockwise shorter than counter-clockwise, participants prefer rising judgments; in the opposite condition, participants prefer falling judgments.

Much uncertainty still exists about the perplexing acoustical phenomena of Shepard tones (also octave-complex or octave-related tones; Shepard, 1964). These tones consist of octave-spaced components, whose amplitudes are generated under a fixed bell-shaped spectral envelope (see Figure 1). This fixed envelope ensures that the different Shepard tones are approximately equal in their over-all loudness (Shepard, 1964). These tones are clearly defined in pitch chroma (e.g., C, Cis, D, …), albeit with octave ambiguities that lead to specific illusions of relative pitch height. Presenting an chromatic sequence of Shepard tones repetitively, the illusion of a continuous rising or falling pitch occurs (similar to the Escher staircase in visual perception), although the sequence repeats physically. Generally, Shepard-tone pairs lead to rising judgments when the clockwise distance of two Shepard tones on the pitch-class circle is shorter than the counter-clockwise distance and lead to falling judgments in the opposite condition, indicating the Gestalt principle of proximity in auditory perception. Shepard alleged that this circularity in relative pitch judgments reflected the circular property of pitch, which is pitch chroma or pitch class. Thus, the pitch-class circle is often used to illustrate Shepard tones (see Figure 2). However, the octave relationship between components is not important in this sort of illusion (e.g., Burns, 1981; Nakajima et al., 1988, 1991). Nevertheless, we use the pitch-class circle because it is a common and well known way to illustrate main aspects of Shepard-tones pitches.

The proximity principle provides no cue for the tritone interval (half-octave, 6 semitones), because the clockwise and counter-clockwise distances are similar to each other (see Figure 1). However, Deutsch (e.g., Deutsch, 1986, 1988) revealed that listeners judge tritone pairs reliably as rising or falling patterns as a function of the position of the tones on the pitch-class circle (tritone paradox, see Figure 2). The tones in the upper half of the circle are heard as higher than those in the lower half of the pitch-class circle. Interestingly, which tones are heard as higher or lower and, thus, the pitch-class-circle orientation varied from person to person, depending on linguistic background (e.g., Deutsch et al., 1987; Deutsch, 1991; Dawe et al., 1998; Giangrande, 1998; Chalikia et al., 2000, 2001; Chalikia and Leinfelt, 2000). Deutsch (1987) and (Deutsch, 1991) revealed systematic differences in the tritone paradox between students raised in California (highest pitch class at D) and students raised in South England (highest pitch class at G). Differences in the tritone paradox for different linguistic backgrounds were also replicated in a Canadian sample (highest pitch class at G, Dawe et al., 1998). Further evidence for correlates was provided by Deutsch et al. (1990), who found a correlation between the tritone paradox and the fundamental frequencies of voice and by Deutsch et al. (2004), who found that mothers and their offspring perceived tritone pairs quite similar. The influence of language on the tritone paradox was also replicated in samples outside the US (Chalikia and Leinfelt, 2000; Chalikia et al., 2001, 2000). In view of the existing literature, evidence exists for individual differences in the tritone paradox based on individual differences in linguistic background. However, some studies failed to reveal systematic differences in subjectively highest Shepard tones for different linguistic backgrounds, for example, Krüger and Lukas (2002), who compared German and Japanese samples and Repp (1994), who compared British, Dutch, and American samples.

Shepard tones of different chroma differ also in their discrete spectral envelope structure. Particularly, the most prominent component, which is characterized by the greatest amplitude within the individual tone compound, varies in frequency and in amplitude (see Figure 1). Particularly, Deutsch (1987) and Repp (1997) discussed whether the tritone paradox is caused by Shepard-tones pitch-classes or is an effect of differences in discrete spectral envelope structure. To separate such spectral envelope effects from pitch-class effects, each tritone pair is constructed under spectral envelopes centered on different positions on the frequency axis. As a result, tritone pairs differed in their discrete spectral envelope structure but not in their pitch classes. With pitch class as the crucial factor, envelope position would hardly affect rising/falling judgments. However, if the spectral structure is the crucial factor, then envelope position would affect rising/falling judgments. In most studies, pitch judgments for most participants were not or only minimally affected by envelope position (Deutsch, 1986, 1987, 1988, 1991; Cohen et al., 1994; Giangrande, 1998; Repp and Thompson, 2010). Some studies, however, found pronounced envelope effects for at least some participants (Repp, 1994, 1997; Krüger and Lukas, 2002; Krüger, 2011). Particularly, Repp (1997) revealed that the subjectively highest Shepard tones were shifted about six semitones from their envelope center. Repp goes on to say that some listener's judgments are swayed by envelope effects whereas others rely more on pitch class. Interestingly, there is an analogous situation for vowel boundary perception where the positioning of harmonics relative to a fixed formant frequency envelope can change sharply as a function of the fundamental frequency (e.g., Hirahara, 1993; Hirahara et al., 1996).

While Deutsch (1991), Deutsch et al. (2004), Deutsch (2012) has explained the tritone paradox by internally presented pitch-class templates, whose orientations lead to the specific individual response patterns in the tritone paradox, other authors have chosen a psycho-acoustic approach and explained the response patterns by the pitch-height ambiguity of Shepard tones (e.g., Terhardt, 1991; Malek, 2013).

Terhardt (1991) in particular explained the tritone paradox by his virtual-pitch theory (VPT, Terhardt et al., 1982a,b, 1986). The VPT postulates that complex tonal stimuli evoke spectral and virtual pitches. Spectral pitches correspond directly to partials whereas virtual pitches do not necessarily (e.g., missing-fundamental tones). For Shepard tones, the most salient virtual pitches correspond to one of the partials (Terhardt et al., 1982b, 1986). Terhardt (1991) suggested that when such virtual pitches form a rising pair, the tritone pair is judged as rising, whereas when they form a falling pair, the tritone pair is judged as falling. Furthermore, virtual pitch salience is determined by, among other factors, the salience of spectral pitches, which depends on the spectral weighting function. This function reaches its maximum at a specific frequency region, the preference region. Terhardt postulated that individual differences in the tritone paradox are possibly due to individual variability in spectral weighting functions. The predictions of VPT for Shepard tones were confirmed by Terhardt et al. (1986) and Repp and Thompson (2010).

Although much research has been carried out on the tritone paradox, only Dawe et al. (1998) have attempted to investigate possible underlying neural processes that may be involved in the tritone paradox. They postulated the involvement of spectral-motion detectors (e.g., Whitfield and Evans, 1965; Shu et al., 1993; Demany and Ramos, 2005; Demany et al., 2009). One type of these cells responds only to ascending frequency patterns, while the other type responds only to descending frequency patterns. The current study aims to provide further evidence for the involvement of spectral-motion detectors in the tritone paradox.

Studies over the past two decades have provided important information on spectral-motion detectors. Single-cell neurophysiological studies on animals revealed that units in the primary auditory cortex respond more consistently to frequency-modulated tones than to steady tones and that some units are tuned to upward and others to downward frequency shifts (e.g., Evans and Whitfield, 1964; Whitfield and Evans, 1965; Mendelson and Cynader, 1985; Weinberger and McKenna, 1988; Mendelson and Grasse, 1992; Mendelson et al., 1993; Zhao and Liang, 1996; Liang et al., 2002; Tian and Rauschecker, 2004; Atencio et al., 2007). Evidence for spectral-motion detectors in humans exists, conducted by MEG (e.g., Sams and Näätänen, 1991; Pardo and Sams, 1993), PET (e.g., Thivard et al., 2000) and fMRI studies (e.g., Hall et al., 2000).

Behavioral methods also supported the idea of spectral-motion detectors. Early adaptation studies revealed that the ability to detect frequency modulations in test stimuli was reduced after exposure to adaptor stimuli with the same frequency modulation rate (e.g., Kay and Matthews, 1972; Regan and Tansley, 1979; Tansley and Suffield, 1983; Lyzenga et al., 2004). Furthermore, Gardner and Wilson (1979) found increased thresholds for the direction of frequency modulations after exposure to adaptor stimuli similar in frequency-modulation direction, indicating the existence of spectral-motion detectors. Holt et al. (2000) showed that frequency-modulated sine-wave glides evoke shifts in vowel categorization, indicating the impact of spectral-motion detectors in speech processing additional to other contextual factors (e.g., Watkins and Makin, 1996; Holt, 1999; Holt and Lotto, 2002; Kluender et al., 2003; Holt, 2006). However, critics argued that these results are not caused by adaptation but by non-sensory sources (for an overview see Altmann and Gaese, 2014) such as a shift of the decision criterion (Wakefield and Viemeister, 1984) or extended training (Moody et al., 1984). However, recent studies provided further support for the spectral-motion-detectors hypothesis. Listeners were able to perceive frequency shifts in nonperceived pitches (Demany and Ramos, 2005; Demany et al., 2009) and streaming judgments on tone sequences (ABA paradigm) were influenced by previous sequences, which can be hardly explained by criterion shift or extended training (e.g., Snyder et al., 2008, 2009).

Dawe et al. (1998) used a spectral-motion adaptation procedure to investigate the involvement of spectral-motion detectors in the tritone paradox. The idea of motion-adaptation procedures in the visual-perception approach is that gazing for some time at an adaptor stimulus moving in one direction fatigues the cells that are selectively tuned to that direction. Afterwards, stationary or ambiguous patterns seem to move in the opposite direction, because the activity in the fatigued cells remains reduced and, hence, the activity in the cells of the opposite direction predominates (motion aftereffect, waterfall effect, e.g., Anstis, 1970).

Evidence exist for motion aftereffects also with auditory stimuli that move in a specific direction in space (e.g., Grantham and Wightman, 1979). Similarly, listening for some time to an adaptor stimulus rising (or falling) in frequency fatigues the cells that are selectively tuned to rising (or falling) frequency shifts in the spectral-motion adaptation procedure. Afterwards, stationary or ambiguous stimuli seem to drift in the opposite frequency direction. Such contrastive aftereffects were shown by several studies (e.g., Shu et al., 1993; Kayahara, 2001; Snyder et al., 2008; Masutomi and Kashino, 2013; Wang and Oxenham, 2014; Alais et al., 2015). Recently, a considerable literature has grown up around the theme of plasticity in auditory cortex and its functional importance for organisms to be flexible (e.g., Fritz et al., 2003; Ulanovsky et al., 2003; Nelken, 2004; Fritz et al., 2005, 2007; Shamma and Fritz, 2014).

Dawe et al. found contrastive aftereffects in the tritone paradox, comparing the response patterns of tritone pairs with and without preceding falling or rising adaptor sequences. Furthermore, they found larger contrastive aftereffects for tritone pairs that begin with Shepard tones in the upper half than for those in the lower half of the pitch-class circle. They suggested that tones in the upper half are differently processed than tones in the lower half. However, the reason for this suggestion remains unclear. A further surprising result was the shift in the individual orientation of the pitch-class circle due to adaptation (+4.5 semitones), indicating a complex underlying process.

The key aspect of the current study is to replicate and extend the Dawe et al. study by revealing that contrastive aftereffects in the tritone paradox are frequency specific. Studies showed that the amount of contrastive aftereffect was significantly reduced when adaptor and test tones were presented in different frequency regions (e.g., Anstis and Saida, 1985; Okada and Kashino, 2003; Snyder et al., 2009; Wang and Oxenham, 2014), indicating that adaptation of spectral-motion detectors in the tritone paradox is also frequency specific. Thus, if spectral-motion detectors are involved in the tritone paradox, given that they are frequency specific, the contrastive aftereffects in the tritone paradox should also be frequency specific. Consequently, reduced contrastive aftereffects are to be expected when the adaptor sequence and the test tritone pair are presented in different frequency regions.

The findings of Dawe et al. (1998) may be somewhat limited by the fact that one cannot rule out a non-sensory explanation. In their study, two groups participated. Both groups first listened to tritone pairs without the preceding sequences. Afterward, the first group listened to tritone pairs preceded by rising adaptor sequences and the second group to tritone pairs preceded by falling adaptor sequences. The observed increase of *lower* responses in the rising group may result from the use of a stricter criterion to report that the second tone was higher after the repeated exposure to the clearly rising Shepard tones. Possibly, participants adopted the clearly rising Shepard tones of the adaptor sequence as their standard for rising patterns. Thus, because the ambiguous tritone pairs differed from this standard, they were less often judged as rising. Similarly, the falling group used a stricter criterion for *lower* responses because they set a standard for falling tones. Another concern is the possible familiarization in the adaptation condition due to the sequential blocked design. In the Dawe et al. study, participants listened first to tritone pairs without adaptor sequences and second, to those with the preceding adaptor sequences. Thus, possibly, the response pattern changed due to repeated listening to the tones and not due to adaptation. Possibly, participants needed time to get used to the very special sounds of Shepard tones.

Thus, the current study uses a within-subject design where control and adaptor conditions are mixed (instead of sequentially presented). It is assumed that criterion shifts do not change several times within an experiment. Furthermore, the concern about possible training or familiarization effects will be ruled out. Furthermore, small methodical problems of Dawe et al. are improved in the current study.

Dawe et al. found no main effect of adaptation, but only an adaptation effect for the Shepard tones located in the upper region of the pitch-class circle, which was possibly due to the limitations of the statistical analysis. One problem was that Dawe et al. used for the statistical analysis the ANOVA with data derived from dichotomous response data, which can lead to serious problems (e.g., Jaeger, 2008). A further problem was possibly that the sample size of 10 participants per group was possibly to small to find main adaptation effects in a between-subject design. Furthermore, Dawe et al. reported differences in the pre- and postadaptation condition descriptively, but reported no statistical analysis. The current study aims to improve this limitation of the Dawe et al. study.

## 2. Methods

### 2.1. Participants

Normal-hearing psychology students from the Martin Luther University Halle-Wittenberg (*n* = 21; 16 women) participated. They were aged on average 23.2 years (*SD* = 3.1). Three participants had no musical experience at the time of the survey; 18 participants had learned at least one instrument or taken singing lessons on average for 5.3 years.

All participants were native German speakers, except from one native Arabic and one native Indonesian speaker. Furthermore, one participant had 2 mother tongues, German and Russian.

Participant recruitment was conducted by notices at the University. The participants were psychology students who need to participate in studies of the psychology department as part of their education. This requirement is approved by the study board at the Department of Psychology, Martin Luther University Halle-Wittenberg. The experiments conducted do not require formal ethical approval according to German Law and the institutional requirements. Before participation the students were informed that the collected data is used in an anonymous form for publication. All students participated voluntarily and were free to opt-out with no contrastive consequences at any time of the experiment. The study was conducted in accordance with the Helsinki declaration and the University Research Ethics Standards.

One participant judged all tritone pairs constructed under the low envelope as falling and all tritone pairs constructed under the high envelope as rising. Thus, the magnitude of effect was zero and it was not possible to extract the highest Shepard tones. Probably, this participant misunderstood the task. His/her data was excluded from the analysis.

### 2.2. Equipment

Participants listened to auditory stimuli via Sennheiser HD 428 headphones. All sounds were generated by MATLAB2011b and presented by the software PxLab (Irtel, 2007). The experiment took place in a semi-soundproof room at the University. The sound pressure level (SPL) of the stimuli was measured by the sound level meter of Center 320 Series Testlink SE-322.

### 2.3. Stimuli

#### 2.3.1. Shepard tones

To compare our data with the results of Dawe et al. (1998), the Shepard tones were constructed according to Dawe et al. (1998). They consisted of six sinusoidal partials separated by an octave. The frequency *f*_*li*_ of the i-th component of the Shepard tone *l* for all *i* = 1, …, *m*, *l* = 1, …, *l*_*max*_ is

(1) $$\begin{array}{l} f_{li}:=f_{l1}\cdot 2^{i-1}, \end{array}$$

where

(2) $$\begin{array}{l} f_{l1}:=f_{\text{min}}\cdot 2^{\left(l-1\right)/l_{\text{max}}}. \end{array}$$

Their amplitudes *a*_*li*_ were determined by a fixed cosine envelope

(3) $$\begin{array}{l} a_{li}\left(f_{li}\right)=0.5-0.5\cdot cos[\frac{2\pi }{m}\cdot log_{\beta }\left(\frac{f_{li}}{f_{\text{min}}}\right)], \end{array}$$

where *m* = 6, β = 2 and *l*_*max*_ = 12. Two sets of Shepard tones were used for adaptor and target stimuli. One were generated under an envelope centered at *A*_3_ (220 Hz, *f*_*min*_ = 27.5), the other were generated under an envelope centered at *C*_6_ (1046.5 Hz, *f*_*min*_ = 130.8125). The duration of the Shepard tones was 500 ms, their sound level was approximately 80 db SPL. At on- and offset, there was of a 53.6 ms sinusoidal ramp. The sample rate was 44,100 Hz.

#### 2.3.2. Control stimuli

The 6 tritone pairs *C#* − *G*, *D#* − *A*, *F* − *B*, *G* − *C#*, *A* − *D#*, and *B* − *F* were formed from Shepard tones of each stimulus set (*A*_3_, *C*_6_).

#### 2.3.3. Adaptor sequences

**Figure 3:**
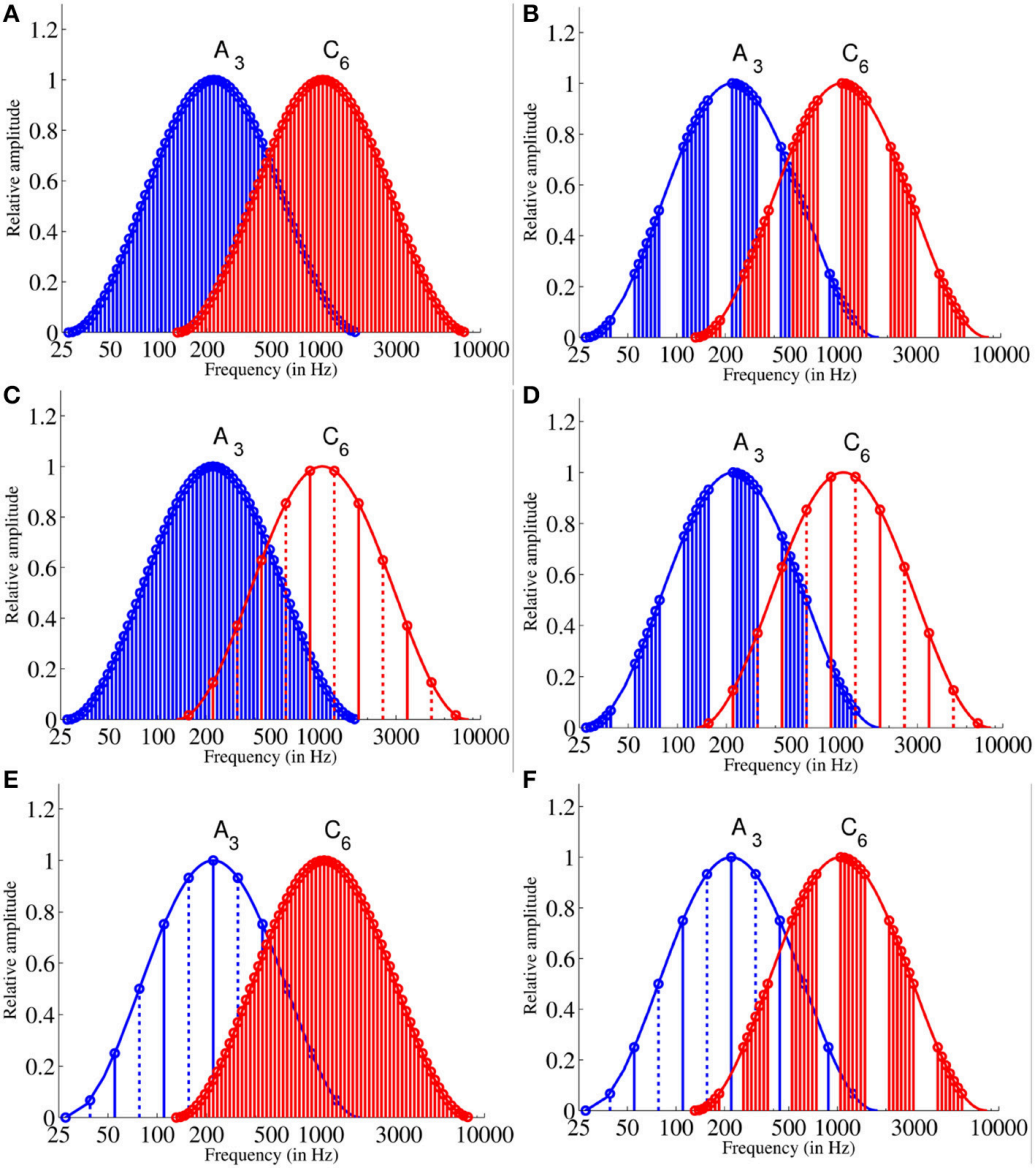
Frequency spectra of adaptor und test stimuli: The 12 Shepard tones of the long adaptor **(A)** and the 7 Shepard tones of the short adaptor sequences **(B)** generated under the *A*_3_ envelope and the *C*_6_ envelope. The stimuli of the long **(C)** and the short **(D)** adaptor sequence generated under the *A*_3_ envelope in comparison to the tritone pair *A* − *D* generated under the *C*_6_ envelope. The stimuli of the long **(E)** and the short **(F)** adaptor sequence generated under the *C*_6_ envelope in comparison to the tritone pair *A* − *D* generated under the *A*_3_ envelope. The solid lines represent the spectra of the first Shepard tones and the dotted lines that of the second Shepard tone. Each Shepard tone consists of 6 octave components. Note, the first or the 6th component is hard to see due to attenuation.

The adaptor sequences were formed out of the 12 Shepard tones *C*, *C#*, *D*, *D#*, *E*, *F*, *F#*, *G*, *G#*, *A*, *A#*, *B* generated under the 2 envelopes (*A*_3_, *C*_6_) using the same method as described above (see Equations 1 and 3) and arranged chromatically clockwise (ascending sequence) or counter-clockwise (descending sequence). The adaptor sequences consisted of six repetitions of the 12 chromatically arranged Shepard tones. The discrete spectral structure of Shepard tones depend on the envelope under that they were generated (e.g., see Figure 3 the tritone pair *A* − *D* generated under *A*_3_ and *C*_6_). Thus, to hold the discrete spectral structure constant over the two envelope sets *A*_3_ and *C*_6_, the ascending adaptor sequences began with the Shepard tone that corresponded to the envelope center (*A* for the *A*_3_ envelope; *C* for the *C*_3_ envelope). The descending adaptor sequences were played in the reversed order and, hence, began with *G#* or *B*, respectively. The adjacent Shepard tones of the sequences were separated by a 100 ms silent interval. The duration was about 43 s. The supplementary files provide examples for the adaptor sequence generated under *A*_3_ (Audio 1) and under *C*_6_ (Audio 2).

#### 2.3.4. Target stimuli

The target stimuli were identical to the control stimuli (the 6 tritone pairs), except that each tritone pair was preceded by 4 s (the first 7 notes) of the adaptor sequence. This short adaptor sequence served as top-up to hold the adaptation relative constant over the test period. The short adaptor sequence and the tritone pair was separated by a 1 s silent interval to prevent grouping effects between target and adaptation stimuli.

Figure 3 shows the frequency spectra of the adaptor sequences and the target stimuli generated under the *A*_3_ and the *C*_6_ envelope. The supplementary files provide examples for the target stimuli (Audios 3–5).

#### 2.3.5. Procedure and research design

The task for the participants were to judge whether the second Shepard tone was *higher* or *lower* in pitch. The participants had as much time as they needed to give their answer. After pressing the answer keys (*higher* or *lower*), the participants got a visual feedback on the screen about their decision. The next trial started after 0.4 s. Participants could have a break as long as they wished at the end of each block.

To exclude the non-sensory explanations discussed above, one should use a complete randomized design instead of the sequential blocked design used in Dawe et al. (1998). However, this would be very time-consuming, because each target stimuli would be preceded ba a 43 s adaptor sequence. Additionally, adaptation effects, which are probably long-lasting, would interfere and extinguish each other between trials. Thus, 12 trials were pooled to one block. The trials within each block and the blocks were randomized (see Figure 5).

**Figure 4:**
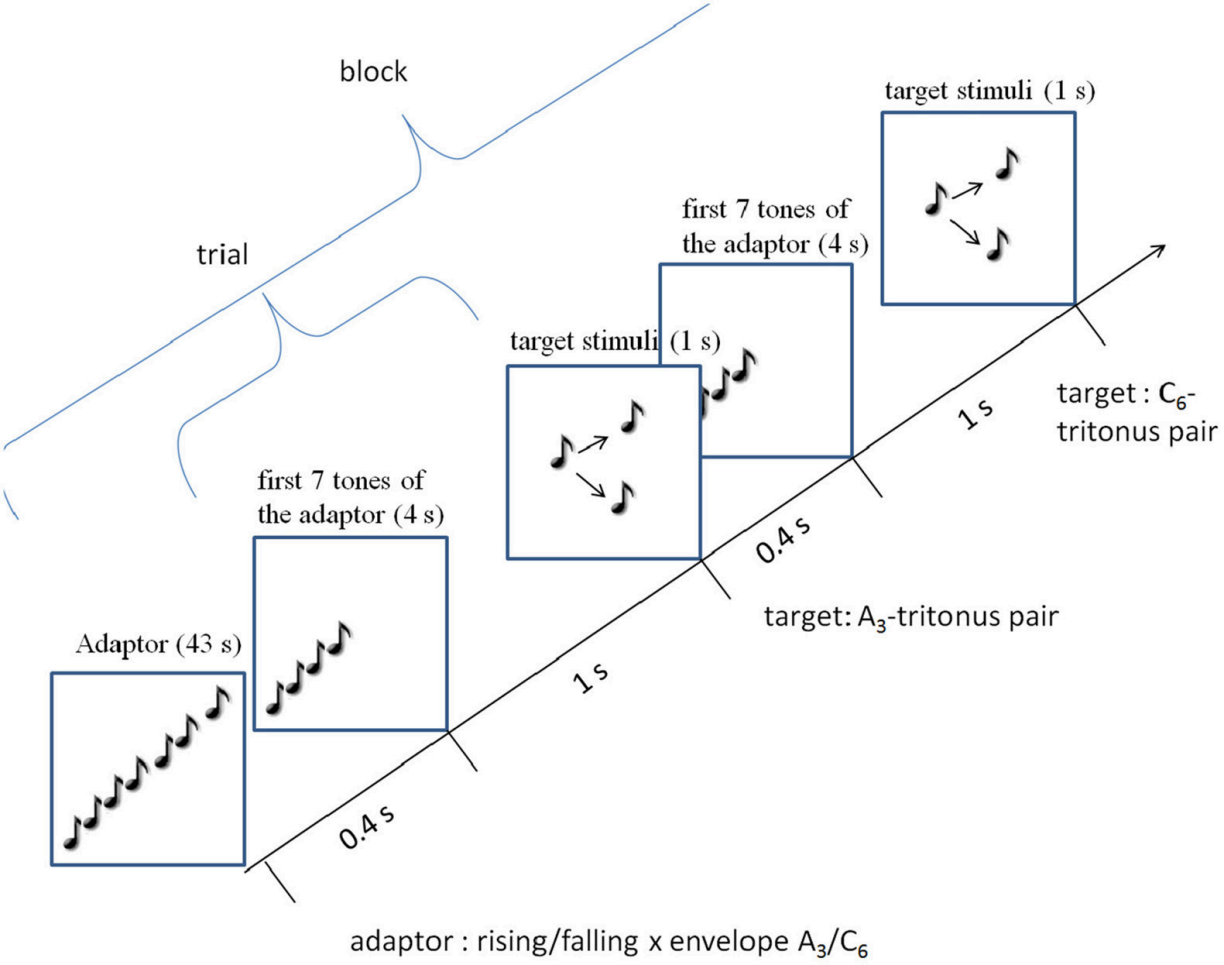
Procedure of an adaptation block: an adaptation block started with a 43 s adaptor sequence (rising/falling × *A*_3_/*C*_6_-envelope). Each trial consisted of the first 7 tones of the adaptor sequence and the target stimuli. Each block contained *A*_3_ and *C*_6_ target stimuli.

There were 5 types of blocks: one control and 4 adaptation blocks (see Figure 4). The control block comprised the 12 control stimuli (the 6 tritone pairs for each envelope). Each of the 4 adaptation blocks comprised one type of adaptor sequences (rising/falling × envelope centers *A*_3_/*C*_6_). Figure 4 shows the procedure of an adaptation block. The adaptation blocks began with the adaptor sequence (43 s), followed by the 12 target stimuli. Each adaptation block comprised only one type of adaptor sequence (e.g., the rising *A*_3_ sequence), but the tritone pairs were generated under the two envelope centers (*A*_3_, *C*_6_), resulting in consistent and inconsistent trials. The consistent trials occurred when tritone pairs and adaptor sequence were generated under the same envelope (*A*_3_ − *A*_3_, *C*_6_ − *C*_6_); the inconsistent condition occurred when tritone pairs and adaptor sequence were generated under different envelopes (*A*_3_ − *C*_6_, *C*_6_ − *A*_3_). Thus, each adaptation block comprised 6 consistent and 6 inconsistent trials.

**Figure 5:**
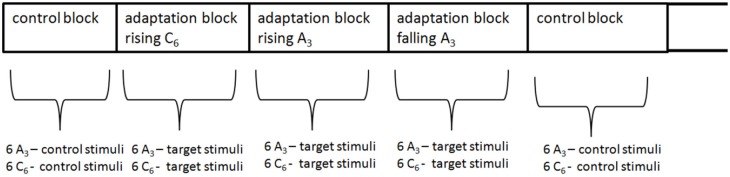
Control and the 4 adaptation blocks were mixed and randomized within the experiment.

Each block was repeated 10-times. Thus, there was 50 blocks and 600 trials. The experiment was split up into two sessions, each lasting about 50 min and was done within 1 or 2 weeks. Each session began with the instruction screen, followed by 3 practice trials.

## 3. Results

### 3.1. Control condition

**Figure 6:**
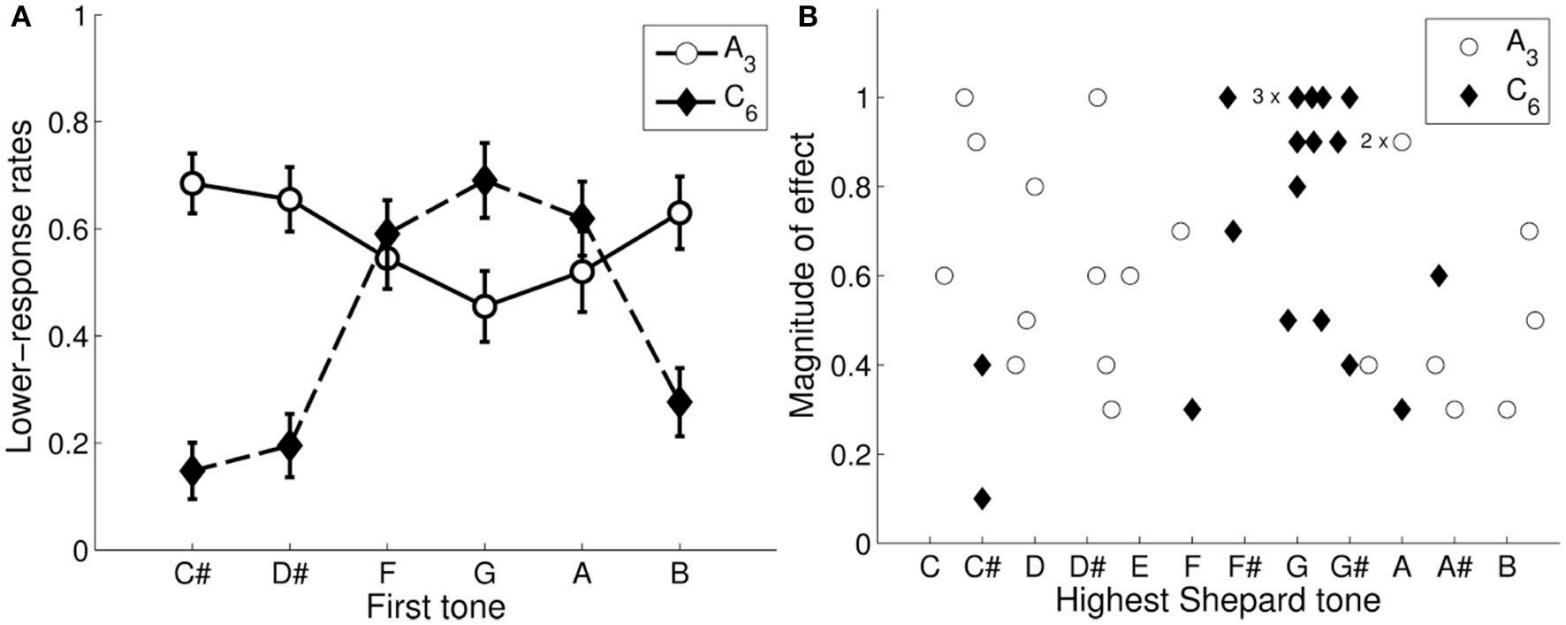
Tritone paradox in the control condition. Averaged lower responses as function of the first Shepard tone generated under the *A*_3_- and the *C*_6_-envelope. The error bars represents the standard error of mean **(A)**. The distribution of the individual highest Shepard tones and magnitudes of effect under the envelope centered at *A*_3_ and *C*_6_ for each participant (**B**, *n* = 20); the magnitude of effect was quantified as the difference of the minimum and the maximum of the individual response function. 2 participants for the *A*_3_-envelope and 3 participants for the *C*_6_-envelope had identical values for the highest Shepard tone and the magnitude of first-tone effect.

*Lower* responses were averaged as a function of the first tone for the 2 envelopes. As can be seen in Figure 6A, the averaged response function for the low envelope (*A*_3_) was less pronounced than that for the high envelope (*C*_6_), mainly because the individual differences were larger in the low than in the high envelope (see Figure 6B). Furthermore, the response functions were roughly inverses of each other (i.e., 6 st removed from each other). Consequently, the Shepard tones *B*, *C#*, *D#*, were judged as higher (i.e., they led to more *lower* responses when forming a tritone pair) than the Shepard tones *F*, *G*, *A* for the tritone pairs generated under the low envelope (*A*_3_); the Shepard tones *F, G, A* were judged as higher than the Shepard tones *B*, *C#*, *D#* for the tritone pairs generated under the high envelope (*C*_6_).

From the individual response functions, the magnitude of effect was quantified as the difference of the minimum and the maximum for each participant. The highest Shepard tones (called also subjectively highest pitch class or peak pitch classes) was set as the point (pitch class) at the response-function peak and was extracted by the procedure described in Repp and Thompson (2010), where the radial angle (rotation angle) of the resultant vector of the data points (*lower* responses) was calculated into semitones relative to C (see also Fisher, 1993). Figure 6B shows the distributions of the magnitude of effects and the highest Shepard tones for the 2 envelope centers.

### 3.2. Aftereffects

#### 3.2.1. Aftereffects in the consistent and inconsistent condition

**Figure 7:**
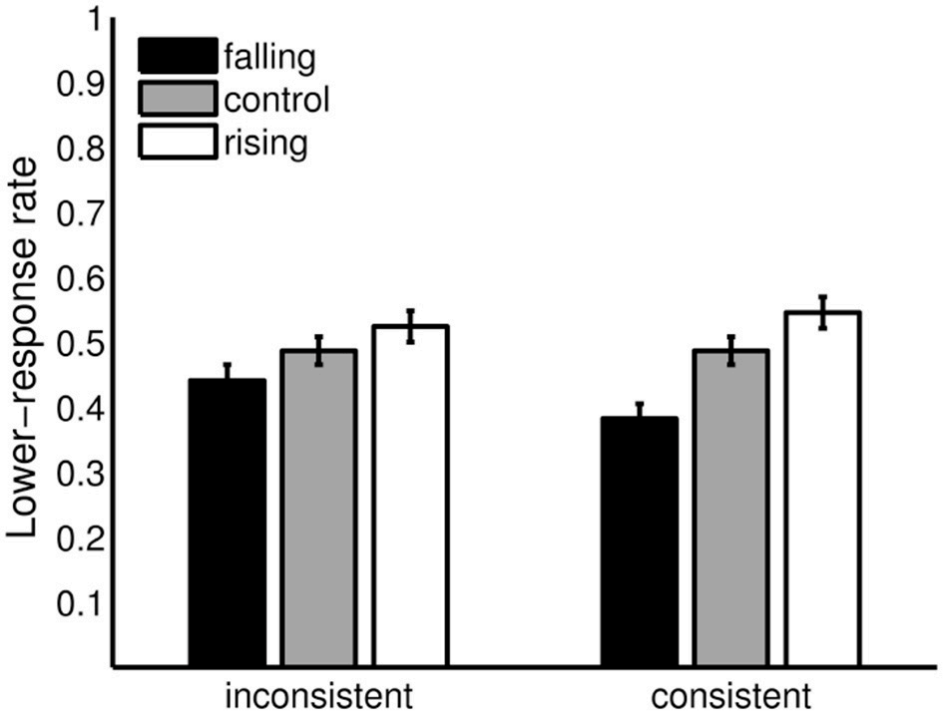
Aftereffects in the consistent and the inconsistent condition. *Lower*-response rates in the inconsistent (envelope centers of the adaptor sequence and tritone pairs *A*_3_ – *C*_6_ and *C*_6_ – *A*_3_) and the consistent condition (envelope centers of the adaptor sequence and tritone pairs *A*_3_ – *A*_3_ and *C*_6_ – *C*_6_) for the falling and rising adaptor sequence in comparison with the control condition. Error bars represents the standard error of mean (*n* = 20).

Figure 7 compares the 2 adaptation conditions (rising/falling adaptor sequences) to the control condition for the consistent and the inconsistent condition. Both adaptor sequences affected *lower* responses in the expected direction in the consistence and in the inconsistent condition. The effects were more pronounced in the consistent than in the inconsistent condition, but for both conditions rather small. The falling adaptor sequence resulted in a decrease (consistent: −0.11, inconsistent: −0.05) and the rising adaptor sequence in an increase (consistent: +0.06, inconsistent: +0.04) of *lower*-response rates in comparison to the control condition.

One-tailed paired Wilcoxons signed-rank tests were used to analyze the adaptation conditions in comparison to the control condition. Parameter-free tests were used, because dichotomous data violated assumptions of the standard procedure. The alpha error was bonferroni-adjusted to 0.0125. Following this criterion, the differences were significant for the consistent condition, falling preceding sequence: *V* = 186.5, *p* = 0.001, *n* = 20 and rising preceding sequence: *V* = 34, *p* = 0.008, *n* = 20, but not for the inconsistent condition, falling preceding sequence: *V* = 141, *p* = 0.034, *n* = 20 and rising preceding sequence: *V* = 53.5, *p* = 0.0494, *n* = 20. However, a trend was also found in the inconsistent condition.

#### 3.2.2. Contrastive aftereffects in low and high shepard tones.

**Figure 8:**
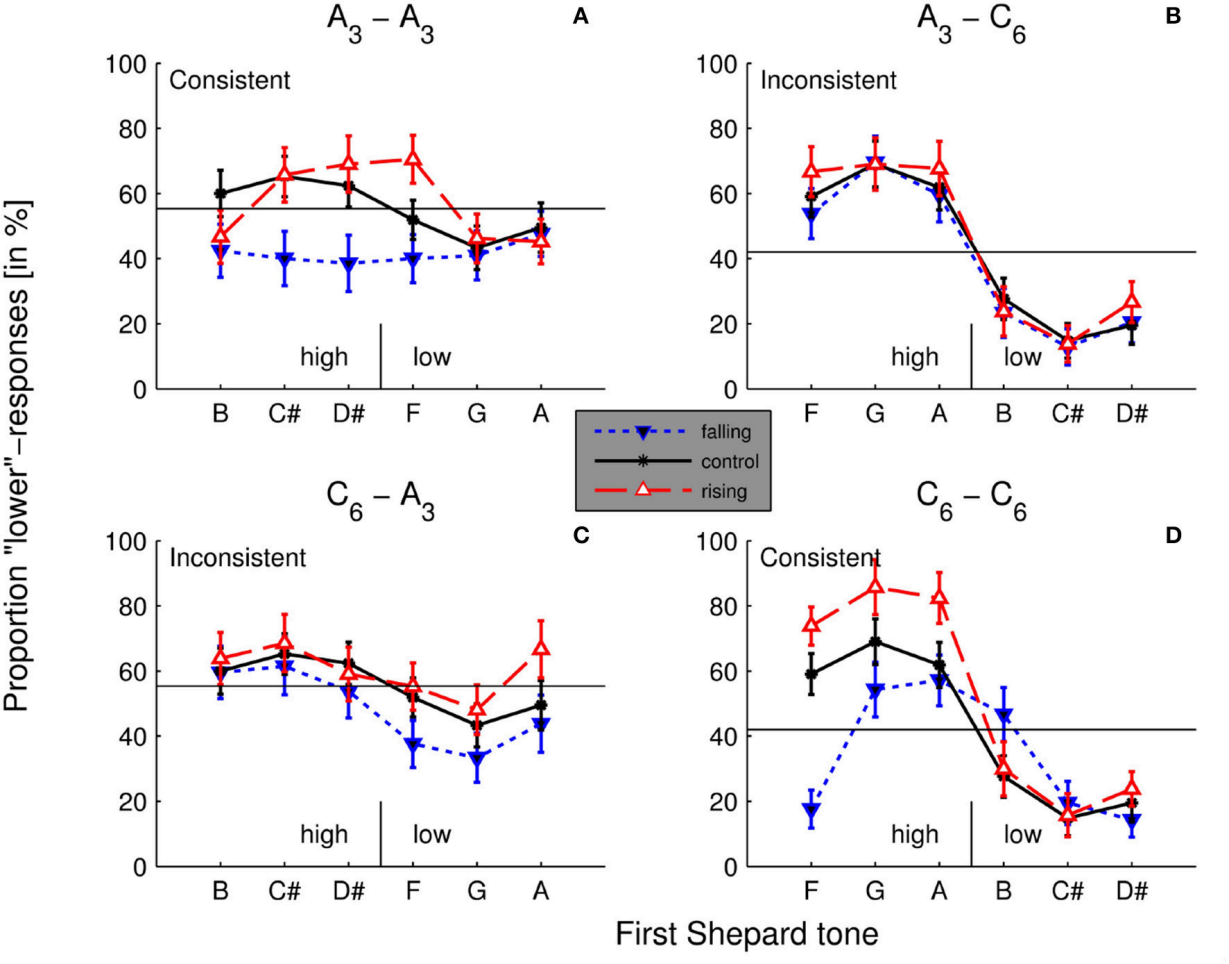
Aftereffects in the different conditions. Averaged percentages of *lower* responses as function of the first Shepard tone in the control condition (without a preceding sequence) and the two experimental conditions (rising/falling preceding sequences) in all 4 adaptor - target combinations *A*_3_ − *A*_3_ **(A)**, *A*_3_ − *C*_6_ **(B)**, *C*_6_ − *A*_3_ **(C)**, *C*_6_ − *C*_6_ **(D)**. The horizontal line depicts the mean of *lower* responses in the control condition. Shepard tones that caused *lower* responses (forming a tritone pair) below the mean were labeled as high, those that caused *lower* responses over the mean as low (in the control condition). Identical envelopes of the tritone pairs and the adaptor lead to the consistent condition and different envelopes to the inconsistent condition (*n* = 20).

As shown in Figure 8, contrastive aftereffects were more pronounced for the high Shepard tones than for the low Shepard tones. The effect was most pronounced when the target and the adaptor stimuli were generated under *C*_6_.

In what follows, aftereffects were quantified as the differences of *lower*-response rates to tritone pairs between control and adaptation blocks. Particularly, aftereffects values should be negative in case of the expected contrastive aftereffects, i.e., the spectral motion of the target stimuli was in the opposite direction to the adaptor stimuli, and positive in case of positive aftereffects. Thus, aftereffect values were quantified as control data minus adaptation data for the rising adaptor sequences, whereas aftereffect values were quantified as adaptation data minus control data for the falling adaptor sequences.

A multi-level (i.e., mixed-effects) logistic regression model (Jaeger, 2008) on aftereffects with the fixed variable consistence (consistent vs. inconsistent), the fixed variable first-tone height (high vs. low), the fixed variable direction of sequence (rising vs. falling) and the random variable participant were conducted. The variable first-tone height was determined by the results in the control condition (see Figure 6). The high Shepard tones were B, C#, D# for *A*_3_-envelope and F, G, A for *C*_6_-envelope, the low Shepard tones were F, G, A for *A*_3_-envelope and B, C#, D# for the *C*_6_-envelope.

The consistence effect, χ^2^(1) = 6.66, *p* < 0.010 and the effect of the first-tone height, χ^2^(1) = 8.91, *p* = 0.003 were confirmed by the analysis. There was a trend of direction, χ^2^(1) = 3.12, *p* = 0.078. Contrastive aftereffects were slightly larger for the falling (.07) than for the rising adaptor (0.05). Furthermore, the interaction effect between the consistence conditions and the first-tone height was significant, χ^2^(1) = 9.48, *p* < 0.002.

**Figure 9:**
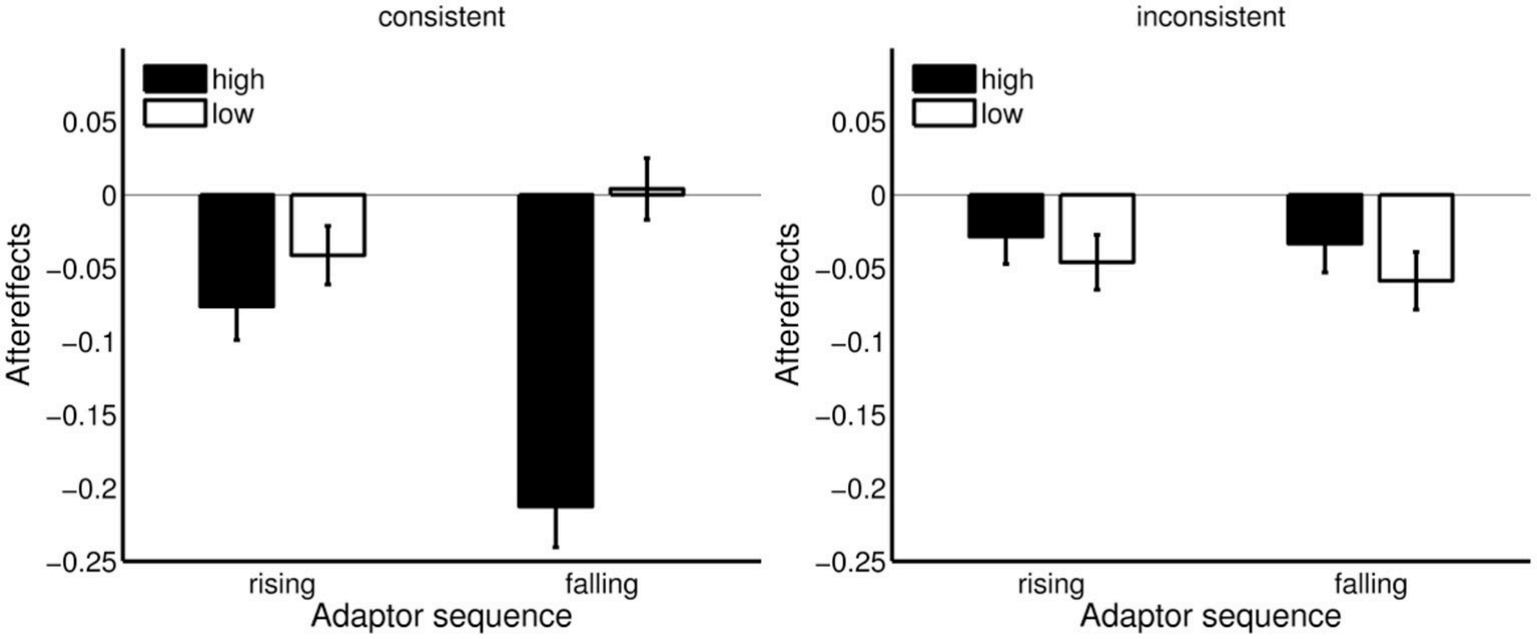
Aftereffects for high and low Shepard tones and rising and falling adaptor sequences in the consistent and inconsistent condition. Rising adaptor sequence: Aftereffects = *lower*-response rate in control condition – *lower*-response rate in adaptation condition; falling adaptor sequence: Aftereffects = *lower*-response rate in adaptation condition – *lower*-response rate in control condition. Error bars represents the standard error of mean (*n* = 20).

As seen in Figure 9, contrastive aftereffects were larger for the high Shepard tones for both adaptor directions in the consistence condition. In contrast, contrastive aftereffects were larger for the low Shepard tones in the inconsistent condition. However, the differences between high and low Shepard tones in the inconsistent condition were much less pronounced than in the consistent condition. No other effect was significant.

The logistic regression model was used because the dichotomous data violated assumptions of the ANOVA. However, results were the same when conducting an ANOVA for repeated measures.

## 4. Discussion

The study found that some contrastive aftereffects in the tritone paradox are frequency specific, which indicates that band-limited spectral-motion detectors play a role in the perception of the tritone paradox. This could occur either by biasing units sensitive to the shifts of the individual frequency components or by biasing those sensitive to shifts of the fundamental frequencies. Either type of biasing could conceivably change the delicate, ambiguous balance of the relative pitch amongst Shepard tones separated by exactly a tritone.

The contrastive aftereffects in the inconsistent condition were nearly half the effects in the consistent condition. However, there were also effects in the inconsistent condition, which was possibly due to the spectral overlap of adapter and target stimuli even in the inconsistent condition. The overlapping frequency range (about 200–1,000 Hz) is just that frequency region that Terhardt (1991) suggested as *preferred region*, which means that pitch matches to Shepard tones correspond to frequencies in that region. Obviously, the overlapping region is crucial for Shepard-tones pitches, explaining the effects in the inconsistent condition. The current study showed that some contrastive aftereffects in the tritone paradox are frequency specific. However, because of the overlapping frequency region, it remains unclear whether all aftereffects are frequency specific. Future research should insert a noise band around 500 Hz to control spectral overlap.

As mentioned before, several studies have revealed evidence for the existence of spectral-motion detectors, which are tuned to upward- or downward frequency shifts. Dawe et al. (1998) revealed contrastive aftereffects and suggested that they were caused by adaptation of spectral-motion detectors, indicating the involvement of these cells in the tritone paradox. The present study has replicated these results using a different research design and statistical analysis, ruling out the possibility that the contrastive aftereffects were due to artifacts in Dawe et al. The current study used randomly mixed adaptation and control conditions instead of the sequential presentation in Dawe et al. Thus, contrastive aftereffects cannot be explained by an overall criterion shift or familiarization effect, which were possible explanations for the results of Dawe et al. because the adaptation blocks followed the control blocks.

The control data revealed that the spectral envelope had an impact on the response patterns, which is in accordance to Repp (1994, 1997). One could suggest that contrastive aftereffects are found because participants judgments mainly rely on spectral cues. However, Dawe et al. (1998) found similar results in a sample, where envelope effects were small.

The results are a bit equivocal given that strong aftereffects were only seen in the consistent high spectral envelope (*C*_6_ − *C*_6_), but not in the consistent low spectral envelope (*A*_3_ − *A*_3_) condition. The typical very pronounced response pattern was not found for the *A*_3_-envelope tones in the control condition due to high individual variability and less pronounced individual patterns. Possibly, the low and dark timber of these tones was very unfamiliar to the participants and caused specific problems to give relative pitch judgments. Adaptation effects were possibly superimposed by such judgment difficulties. Another possibility is that the *A*_3_ adaptor was less effective than the *C*_6_ adaptor. Participants were instructed to ignore the adaptor. Subjectively, when listen to the sequences, this seems to be easier for the *A*_3_ than the *C*_6_ adaptor, because the pitches of *A*_3_ seems less salient than those of *C*_6_. Rezec et al. (2004) showed that attention can increase the motion aftereffect in the visual system. Attention effects would also explain why the *C*_6_ adaptor, but not the *A*_3_ adaptor, had an effect in the inconsistent condition.

The contrastive aftereffect revealed in the present study could also be interpreted as the context effects revealed by several studies (Repp, 1997; Giangrande et al., 2003; Englitz et al., 2013; Chambers and Pressnitzer, 2014; Chambers et al., 2017). Repp (1997) found that the principle of proximity is not restricted to tone pairs, but also applies to triplets. Specifically, participants have judged the tritone pair *C* − *F#* more often as rising when it was preceded by the Shepard tone *D#* (3 semitones clockwise or 9 semitones counter-clockwise with respect to the first tone). When it was preceded by *A* (9 semitones clockwise or 3 semitones counter-clockwise with respect to the first tone), the reversed pattern occurred. Repp explained this result by the fact that in both cases the total range was kept to 6 semitones. More recent studies have revealed context effects caused by preceding tone sequences (Englitz et al., 2013; Chambers and Pressnitzer, 2014; Chambers et al., 2017). Preceding tones have caused upward biases when they have been shifted less than 6 semitones from the first tone (clockwise). Preceding tones have led to downward biases when they have been shifted more than 6 semitones from the first tone. In the present study, the 7-note short adaptor sequences before each tritone pair might have served as contextual upward or downward bias.

To discuss this point, we consider the consistent condition *C*_6_ − *C*_6_ more closely because of the pronounced contrastive aftereffects. The short version of the rising *C*_6_ sequence comprised the Shepard tones *C*, *C#*, *D*, *D#*, *E*, *F*, *F#*, and *G*. Possibly, these tones (except for *C*) caused an upward bias for the tone pair *C#* − *G* because the clockwise distances the sequence tones were shifted from the first Shepard tone were shorter than the counter-clockwise distances. In contrast, the sequence tones caused a downward bias for the tone pair *G*-*C#*, because the reverse pattern occurred. Thus, the prior context would cause fewer *lower* responses in *C#* − *G* and would cause more *lower* responses in *G* − *C#*. Actually, there were more *lower* responses for *G* − *C#*, but not fewer *lower* responses in *C#* − *G* in the study data. Thus, the response pattern for the rising *C*_6_ adaptor sequence cannot be explained by assuming that only the prior context was active. Thus, possibly, adaptation and context effects were active. For this, the effects would add up in *G* − *C#* and would cancel each other in *C#* − *G*, which is in accord with the study data.

All in all, prior context effects cannot be excluded, but also cannot explain the present results. Possibly, the different contrastive aftereffects for the high and the low Shepard tones were caused by a complex interaction between such context and adaptation effects. However, this suggestion seems unlikely given that Dawe et al. (1998) found similar results despite using different prior contexts and envelope centers. In future research, a control condition that comprises prior context without a rising or falling melody should be preferred to a control condition without a prior context.

Furthermore, the present study has confirmed the result of Dawe et al. (1998) that contrastive aftereffects were larger for the high than for the low Shepard tones, supporting the sensory adaptation explanation against the non-sensory explanation, because a criterion shift would be uniform for all tritone pairs. Surprisingly, the increased contrastive aftereffects in consistent trials were only found for the high but not for low Shepard tones, supporting Dawe et al. suggestion that high and low Shepard tones are processed differently. Little is known about the differences in high and low Shepard tones, but it might be related to language phenomena, given that the tritone paradox is linked to the language domain (e.g., Deutsch, 1991). Recently, a considerable literature has grown up that show the connection between music and language (see for review: Tillmann, 2014; Peretz et al., 2015). Differences in pitch correspond to differences in word meaning in tone but not in non-tone languages. Tone-language (e.g., Mandarin, Cantonese, Vietnamese) speakers had enhanced pitch-perception abilities compared to English or French (non-tone languages) speakers (Pfordresher and Brown, 2009; Hove et al., 2010; Wong et al., 2012; Bidelman et al., 2013). Furthermore, there is a higher prevalence of absolute pitch listeners (i.e., persons who are able to name tones (e.g., *E*_5_) without a reference tone) among tone-language than among non-tone language speakers (e.g., Deutsch et al., 2006, 2009). Further evidence came from persons with congenital amusia, which have impaired pitch processing. These persons have also deficits in processing speech intonation (e.g., falling pitches for statements, rising pitches for questions) in their mother-tongue (e.g., Patel et al., 2008; Liu et al., 2010). High Shepard tones form falling patterns and low Shepard tones form rising patterns. Thus, the differences in contrastive aftereffects for high and low Shepard tones are possibly due to differences in rising/falling patterns. Rising and falling intonations differ in their function in spoken language (e.g., Cruttenden, 1981; Gussenhoven, 2002); falling intonation are used for statements, rising intonation for questions, for example. Interestingly, Snow (1998) showed that 4-year-old children do only imitate the adult-modeled falling but not rising pitch contour, possibly because the rising contour needs more effort than the falling or different motor demands are associated with the production of rising and falling contours (Xu and Sun, 2002) Thus, possibly, different mechanisms for the detection of rising and falling patterns have developed. Thus, a further understanding of the underlying mechanism of Shepard tones may help to further knowledge of the underlying process in language perception.

## Author contributions

All authors contributed to the study idea and design. SM implemented the computer code. KS conducted the research and collected all the data. All authors analyzed and interpreted the data. SM prepared the draft manuscript, KS provided critical revisions. All authors approved the final version of the manuscript for submission.

### Conflict of interest statement

The authors declare that the research was conducted in the absence of any commercial or financial relationships that could be construed as a potential conflict of interest.
